# Smoking, Alcohol Consumption, and Risk of Arterial Stiffness: A Two-Sample Mendelian Randomization Study

**DOI:** 10.31083/j.rcm2507255

**Published:** 2024-07-09

**Authors:** Yingzhen Gu, Zuozhi Li, Xiaorong Han, Jinxing Liu, Yifan Li, Wei Zhang, Naqiang Lv, Aimin Dang

**Affiliations:** ^1^Fuwai Hospital, National Center for Cardiovascular Diseases, Chinese Academy of Medical Science and Peking Union Medical College, 100037 Beijing, China

**Keywords:** smoking, alcohol consumption, arterial stiffness, Mendelian randomization

## Abstract

**Background:**

While observational studies have demonstrated connections between cigarette smoking, alcohol consumption, and arterial stiffness, establishing a causal relationship has proven challenging because of potential confounding factors. To address this problem, we employed a two-sample Mendelian randomization approach.

**Methods:**

We selected genetic instruments for these risk factors from genome-wide association studies encompassing 3,383,199 individuals at the genome-wide significance level (*p* < 5 × ${\mathrm{10}}^{-9}$). Arterial stiffness data were acquired from the UK Biobank, which included 127,121 participants. Our primary analysis utilized the inverse variance-weighted method to explore causality. To confirm our results’ robustness, we conducted sensitivity analyses using Egger regression, the weighted median method, and Mendelian Randomization Pleiotropy RESidual Sum and Outlier (MR-PRESSO).

**Results:**

Our analysis revealed a significant association between genetic inclination to smoking initiation and an increase in the arterial stiffness index (β = 0.11; 95% confidence interval [CI], 0.06 to 0.16; *p* = 1.95 × ${\mathrm{10}}^{-5}$). Additionally, there was a suggestive connection between genetically predicted number of cigarettes per day and the arterial stiffness index (β = 0.05; 95% CI, 5.25 × ${\mathrm{10}}^{-4}$ to 0.10; *p* = 4.75 × ${\mathrm{10}}^{-2}$). No causal relationships were observed between the genetically predicted age of smoking initiation, smoking cessation, or alcohol consumption and the risk of arterial stiffness index.

**Conclusions:**

This Mendelian randomization study indicates that smoking initiation is likely a causative risk factor for arterial stiffness. However, further research is needed to determine if the quantity of daily cigarettes directly contributes to arterial stiffness development. Regarding alcohol consumption, age of smoking initiation, and smoking cessation, there was insufficient evidence to establish causality.

## 1. Introduction

Arterial stiffness is an important hallmark of early vascular aging and an independent risk factor for cardiovascular diseases and overall mortality, after adjusting for traditional risk factors [1, 2, 3, 4, 5]. The arterial stiffness index, a convenient noninvasive measure of arterial stiffness, is determined by recording digital volume waveforms through infrared finger sensors, specifically employing photoplethysmography [6]. This index has demonstrated robust associations with alternative methods of assessing arterial stiffness, notably a correlation of r = 0.58 (*p* < 0.01) with pulse wave velocity and r = 0.80 (*p* < 0.01) with the augmentation index [7].

Previous investigations have highlighted smoking as a significant contributor to arterial stiffness, with smoking cessation being shown to partially reverse the impact on arterial stiffness [8, 9]. However, data on the influence of alcohol consumption on arterial stiffness remains inconclusive [10, 11]. Although evidence of smoking and alcohol use on arterial stiffness has been obtained mainly from observational studies, potential confounders and other unmeasurable biases may undermine the real causality.

While it is not easy to perform randomized controlled trials because of ethical issues and high cost, Mendelian mendelian randomization (MR) represents an innovative analytical strategy to address these issues. This technique utilizes genetic variants associated with exposures as instrumental variables to assess the causal links between these exposures and health-related outcomes [12]. Given that the genetic code is randomly assigned and remains constant from conception, MR studies are less susceptible to unmeasured confounding variables than traditional observational studies, helping to mitigate the impact of reverse causality. Therefore, we employed a two-sample MR approach to examine the potential causal relationships between smoking, alcohol consumption, and risk of arterial stiffness.

## 2. Methods

### 2.1 Research Design

**Fig. 1.**
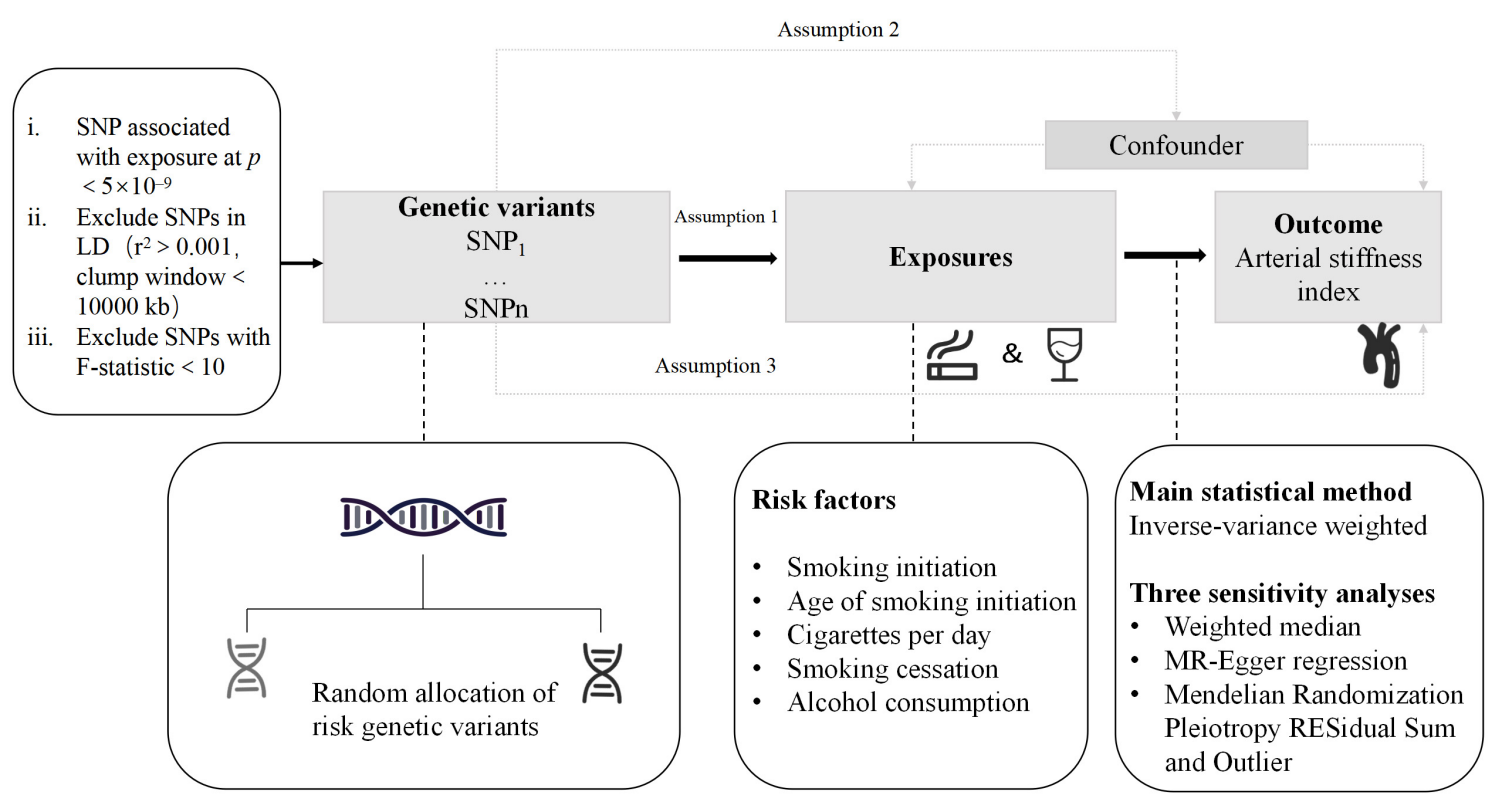
**Overview of the study design.** SNP, single-nucleotide polymorphism; LD, linkage disequilibrium.

Three critical assumptions for this MR study are outlined in Fig. 1. First (i), the relevance assumption requires that single-nucleotide polymorphisms (SNPs) employed as instrumental variables should exhibit a robust association with exposure to the phenotypic variable [13, 14]. Second (ii), the independence assumption stipulates that the selected SNPs should not be correlated with any potential confounding factors [13, 14]. Third (iii), the exclusion restriction assumption requires that the selected SNPs should impact the outcome exclusively through the exposure, without involving any alternative pathways [13, 14].

### 2.2 Data Source for Arterial Stiffness

We obtained summary statistics for arterial stiffness, as indicated by arterial stiffness index, from a meta-analysis of a genome-wide association study (GWAS) involving participants of European descent from the UK Biobank (n = 127,121, age 56 ± 8.1, 48.1% males) [15]. This study utilized photoplethysmography to capture the digital volume pulse, visualized as a dicrotic waveform. The arterial stiffness index was derived by recording the interval between the peaks of the direct and reflected components [6]. The GWAS analysis was adjusted for various factors, including age, age squared, sex, weight, device used for pulse waveform acquisition, smoking status, mean arterial pressure, and the first ten principal components.

### 2.3 Selection of Genetic Instruments

The genetic instrumental variants associated with smoking and alcohol consumption were selected from the latest and most extensive GWAS meta-analysis conducted by the GWAS and Sequencing Consortium of Alcohol and Nicotine Use (GSCAN). This meta-analysis involved approximately 60 cohorts, representing individuals from four major global ancestry clines, with a predominant European representation of approximately 79% [16]. Smoking initiation phenotypes comprised two variables: a binary phenotype indicating whether an individual had ever smoked regularly (SmkInit; n = 3,383,199) and the age when the individual commenced regular smoking (AgeSmk; n = 728,826). The intensity of smoking among former and current regular smokers was assessed using cigarettes per day (CigDay; n = 784,353). Smoking cessation was defined as a binary phenotype contrasting former smokers with current smokers (SmkCes; n = 1,400,535). Alcohol consumption was quantified as drinks per week, regardless of the type of alcohol consumed (DrnkWk; n = 2,965,643) [17].

To reduce potential bias, we selected genetic instruments markers from only subjects of European ancestry that reached the genome-wide significance level (*p* < 5 × ${\mathrm{10}}^{-9}$) in the respective GWAS. Linkage disequilibrium (LD) among SNPs was estimated by referencing the 1000 Genomes European panel [18]. SNPs in LD ($r^{2}$ > 0.001) within a clump window of less than 10,000 kb were excluded. We employed F statistics to assess the robustness of the association between each instrumental variant and the exposure. The F statistics were computed using the following formula: F statistics = β^2^/${\mathrm{SE}}^{2}$. Generally, F < 10 indicates that the instrumental variables used are more vulnerable to weak instrument bias [19].

### 2.4 Statistical Analysis

Considering the different degrees of heterogeneity among SNP effects, we employed either random effects or fixed effects in the inverse variance weighted (IVW) model as the primary statistical method, which provides the greatest statistical power [20, 21]. However, it is worth noting that the IVW model yields a weighted average of SNP effects with the constraint that the intercept is set to zero, increasing vulnerability to potential issues such as invalid instrument bias or pleiotropy. To enhance the robustness of the results from the IVW method, we further performed a suite of other well-established MR sensitivity analyses. These included MR–Egger regression [22], the weighted median method [23], and MR-PRESSO [24]. MR–Egger regression estimates causation from weighted regression and uses the average pleiotropy effect as the intercept but is prone to be less precise [25]. In addition, the *p* value for the MR–Egger intercept test was used to indicate the presence of pleiotropy [22]. The weighted median method, which assumes that at least half of the SNPs are valid instruments, is more sensitive to outliers [23]. The MR-PRESSO can identify outliers and provide an estimate following their exclusion. Given that these methods are based on distinct assumptions, the consistency of effects observed across multiple methods strengthens our ability to draw more compelling causal conclusions.

Cochran’s Q statistic in IVW estimators was estimated to check for evidence of heterogeneity across individual SNPs. A *p* value < 0.05 suggested strong heterogeneity among these instrumental variables. We used the random effects IVW model to verify causal associations; otherwise, the fixed effects model was used. To address the issue of multiple testing, we implemented a Bonferroni correction by setting a 2-side significance level of 0.01 (0.05 divided by the 5 risk exposures). Associations with *p*-values < 0.01 were considered statistically significant, while those with *p*-values ≥ 0.01 and ≤ 0.05 were deemed suggestive. All analyses were performed using the “TwoSampleMR” and “MRPRESSO” packages within R version 4.3.0 (R Foundation for Statistical Computing, Vienna, Austria).

## 3. Results

This study analyzed data from a genome-wide association study (GWAS) on 127,121 individuals of European descent from the UK Biobank, with an average age of 56 (±8.1 years) and 48.1% being male [15]. We identified a total of 891 SNPs, with 460 related to smoking initiation (SmkInit), 23 related to the age of smoking initiation (AgeSmk), 90 related to the number of cigarettes consumed per day (CigDay), 77 related to smoking cessation (SmkCes), and 241 and related to the number of drinks consumed per week (DrnkWk) as shown in Table 1.

**Table 1.** **Genetic predictors of risk factors and their impact on the arterial stiffness index**.

Exposures	SNP	F statistics	Cochran’s Q	$P_{\text{cochrane’s Q}}$	$P_{\text{pleiotropy}}$	MR–Egger			Weighted median			MR-PRESSO		
						β	95% CI	*p*	β	95% CI	*p*	*p*	Outliers	$P_{\text{distortion}}$
SmkInit	460	34–767	586.29	1.90 × ${\mathrm{10}}^{–\,6}$	0.97	0.11	–0.05, 0.27	0.17	0.07	–4.49 × ${\mathrm{10}}^{–\,3}$, 0.14	0.07	1.49 × ${\mathrm{10}}^{-5}$	0	NA
AgeSmk	23	17–89	52.91	2.34 × ${\mathrm{10}}^{–\,4}$	0.10	0.52	–0.11, 1.14	0.12	0.17	–0.03, 0.38	0.10	0.74*	2	0.85
CigDay	90	34–1837	93.76	0.22	0.84	0.04	–0.06, 0.14	0.42	0.05	–0.03, 0.13	0.22	0.08	0	NA
SmkCes	77	34–519	95.49	0.05	0.57	0.08	–0.18, 0.35	0.53	4.14 × ${\mathrm{10}}^{–\,3}$	–0.14, 0.14	0.95	0.86	0	NA
DrnkWk	241	34–1301	346.70	1.53 × ${\mathrm{10}}^{–\,6}$	0.62	–0.01	–0.14, 0.12	0.87	–0.06	–0.18, 0.05	0.29	0.20*	3	0.86

Abbreviations: MR, Mendelian Randomization; CI, confidence interval; SmkInit, smoking initiation; AgeSmk, age of smoking initiation; CigDay, cigarettes per day; SmkCes, smoking cessation; DrnkWk, drinks per week; NA, not available; SNP, single-nucleotide polymorphism; IVW, inverse variance weighted; MR-PRESSO, Mendelian Randomization Pleiotropy RESidual Sum and Outlier. $P_{\text{cochrane’s Q}}$ is the *p* value for Cochran’s Q statistic in IVW estimators, and a *p* value < 0.05 suggested strong heterogeneity. $P_{\text{pleiotropy}}$ is the *p* value obtained from the MR–Egger intercept test, where a *p* value < 0.05 signifies the presence of significant horizontal pleiotropy. $P_{\text{distortion}}$ is the *p* value acquired from the MR-PRESSO distortion test, where a *p* value < 0.05 indicates significant disparities between estimates before and after the removal of outliers. * indicates the *p* value obtained from MR-PRESSO after outlier removal.

**Fig. 2.**
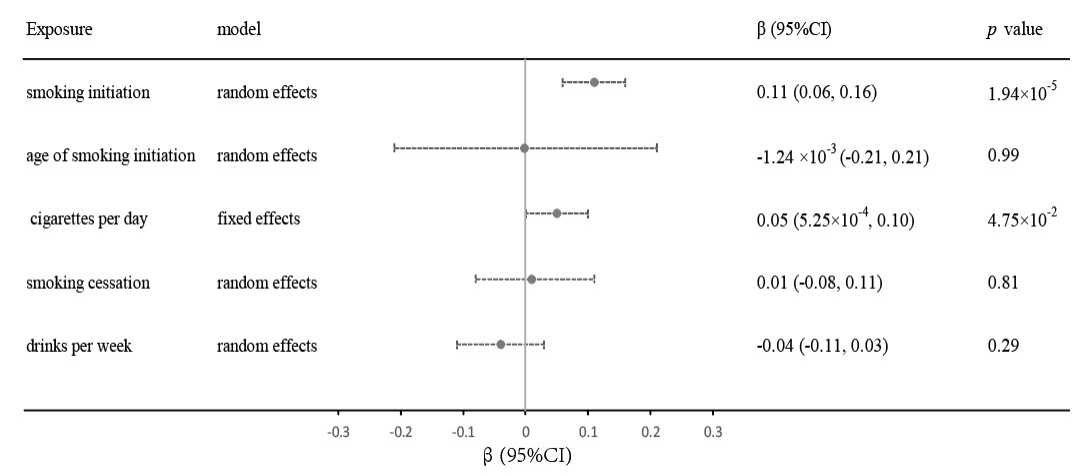
**Relationships between genetic factors and the risk of arterial stiffness index.** Estimates were obtained using the IVW method, and the selection between random effects or fixed effects was determined based on the extent of heterogeneity. CI, confidence interval; IVW, inverse variance weighted.

Our findings, using the IVW method, revealed a significant link between genetic predisposition to smoking initiation and increased arterial stiffness (β = 0.11; 95% confidence interval [CI], 0.06 to 0.16; *p* = 1.95 × ${\mathrm{10}}^{-5}$). We found a suggestive association between genetic prediction of CigDay and arterial stiffness index risk (β = 0.05; 95% CI, 5.25 × ${\mathrm{10}}^{-4}$ to 0.10; *p* = 4.75 × ${\mathrm{10}}^{-2}$). In contrast, there were no causal associations between genetic predictions for AgeSmk (β = –1.24 × ${\mathrm{10}}^{-3}$; 95% CI, –0.21 to 0.21; *p* = 0.99), SmkCes (β = 0.01; 95% CI, –0.08 to 0.11; *p* = 0.81), or DrnkWk (β = –0.04; 95% CI, –0.11 to 0.03; *p* = 0.29), and the arterial stiffness index (Fig. 2).

There were no consistent positive results for SmkInit, CigDay, or arterial stiffness index according to the weighted median or MR–Egger test (Table 1). However, the findings from the MR-PRESSO method consistently indicated that a genetic predisposition to SmkInit was positively associated with arterial stiffness index (β = 0.11; *p* = 1.45 × ${\mathrm{10}}^{-5}$) (Table 1).

Sensitivity analyses using the MR–Egger intercept showed no evidence of horizontal pleiotropy—where genetic variants might influence the outcome through pathways other than the exposure of interest—for any of the SNPs under investigation (all *p* > 0.05) (Table 1). Except for the genetic instruments used for CigDay, Cochran’s Q statistic demonstrated significant heterogeneity among the SNP effects (all *p* < 0.05), as shown in Table 1. The results remained consistent ($P_{\text{distortion}}$> 0.05) after removing potential outliers through MR-PRESSO, as detailed in Table 1. Additionally, the strength of the genetic instruments was validated by F statistics for the instrumental variants, all of which were above the threshold of 10, showcasing their robustness. This information can be found in Table 1.

## 4. Discussion

To the best of our knowledge, the present study is the first to analyze the potential causal links between smoking and alcohol consumption and between smoking and the arterial stiffness index by using the MR framework. Our findings provide robust evidence that a genetic predisposition to smoking initiation is correlated with an elevated risk of arterial stiffness. In addition, there was a suggestive association between the number of cigarettes per day and an elevated risk of arterial stiffness. Nevertheless, we found no significant causal associations between risk factors for the age of smoking initiation, smoking cessation or weekly alcohol consumption, and arterial stiffness. These results suggested that cigarette smoking might be a causal risk factor for arterial stiffness. Based on analytical results involving different smoking phenotypes, this study highlighted that prevention of smoking, rather than cessation can help reduce the risk of arterial stiffness.

Many studies have suggested that smoking is strongly associated with arterial stiffness. Recently, Hahad *et al*. [26] reported a robust and dose-dependent correlation between smoking and increased arterial stiffness in a study of 15,010 participants, regardless of sex. Using pulse wave velocity as the measurement method of arterial stiffness, George *et al*. [27] conducted a prospective, randomized control trial in 145 smokers without established cardiovascular disease, and reported that there was a significant improvement in vascular stiffness within one month of switching from tobacco cigarettes to electronic cigarettes. In a prospective study of 2054 Japanese subjects with a 5- to 6-year follow-up, Tomiyama *et al*. [28] reported that continuous smoking could accelerate the age-related increase in arterial stiffness in larger to middle-sized arteries. Their study also established a clear dose‒response relationship between the quantity of cigarettes consumed and the accelerated progression of arterial stiffness. However, unlike the above studies that identified a dose-dependent association between arterial stiffness and smoking heaviness, Schmidt *et al*. [29] did not find any associations between cigarettes per day and pulse wave velocity. Additionally, the impact of smoking cessation on arterial stiffness has not been sufficiently investigated. Most existing studies have shown improvements in arterial stiffness with smoking cessation [8, 30], and even vascular dysfunction can be reversed with nicotine replacement therapy [31]. In contrast, some studies have suggested that arterial stiffness caused by chronic smoking might be irreversible even after smoking cessation [29, 32, 33]. Schmidt *et al*. [29] suggested that the positive associations between smoking cessation and arterial stiffness might not carefully consider the role of longitudinal weight gain and changes in blood pressure that accompany smoking cessation. Little attention has been given to the association between the age of smoking initiation and arterial stiffness. Our analysis further provided evidence that smoking initiation was detrimental to arterial stiffness and that the number of cigarettes smoked per day might influence the acceleration of arterial stiffness, while smoking cessation and age at smoking initiation might not be associated with arterial stiffness.

The mechanisms by which smoking worsens arterial stiffness are complex. Smoking-induced vascular endothelial dysfunction plays an important role in arterial stiffness. A growing body of evidence suggests that smoking disrupts endothelial homeostasis by diminishing the bioavailability of nitric oxide [34]and increasing the incidence of circulating proinflammatory agents [35, 36]. Importantly, nitric oxide and endothelium-derived hyperpolarizing factors regulate arterial stiffness [37]. Moreover, insulin resistance induced by cigarette smoking accelerates the development of arterial stiffness [38, 39, 40].

The impact of alcohol consumption on arterial stiffness remains a debated issue. Nakanishi *et al*. [41] observed a dose‒response relationship between alcohol consumption and increased arterial stiffness without adjusting for confounders at baseline over a 9-year follow-up period. A systematic review of 13 studies provided preliminary evidence that light-to-moderate alcohol consumption was associated with improvements in arterial stiffness values, whereas high doses accelerated arterial aging [10]. However, a recent study of 153 healthy, nonsmoking, nonobese individuals did not identify a significant association between arterial stiffness and alcohol consumption [42]. In an analysis of a cross-sectional study involving 43,946 individuals aged 35–75 years, Gonzalez-Sanchez *et al*. [43] identified a J-shaped association between alcohol consumption and markers of vascular structure, including carotid intima-media thickness and arterial stiffness, as measured by carotid-femoral pulse wave velocity values. However, they did not observe significant associations with brachial-ankle pulse wave velocity or cardio-ankle vascular index, two alternative methods for assessing arterial stiffness. Considering the equivocal findings, our study suggested that alcohol consumption has no significant positive associations with arterial stiffness.

The major strength of this study is that the causality was based on genetic instrumental variables, which reduces the influence of unmeasurable confounders and makes reverse causality less likely. Additionally, the genetic variants used in this study were derived from the most recent meta-analysis of more than 60 cohorts comprising 3.4 million participants, and the outcome datasets of the arterial stiffness index were obtained from a large-scale GWAS meta-analysis including 143,590 individuals, which yielded adequate statistical validity to estimate causality.

Inevitably, there are several limitations. Because of the current summary level of two-sample MR analysis, we were unable to examine potential nonlinear relationships and therefore could not explore the potential J- or U-shaped associations between risk factors and arterial stiffness. Moreover, the weighted median and MR–Egger methods yielded results that were inconsistent with those obtained from the IVW method, which partially compromised the robustness of the causal relationships. Nonetheless, MR-PRESSO provided consistent results, and no horizontal pleiotropy was found. Considering the comparison of statistical power and the rationale of the different methods, the results of the IVW method were more convincing. Furthermore, there was a degree of sample overlap in the MR analysis, as the GWAS meta-analysis for genetic instruments included data from the UK Biobank. Nevertheless, given the substantial power of most of our analyses, this overlap may not have a significant impact on the results. Finally, despite the large sample size, the subjects were predominantly of European descent. This raises questions about the generalizability of the findings to other groups and populations.

## 5. Conclusions

This MR analysis suggests that a genetic predisposition to smoking initiation plays a causal role in increasing the risk of arterial stiffness. However, further research is warranted to explore whether the number of cigarettes smoked per day has a direct causal effect on arterial stiffness. As for alcohol consumption, age of smoking initiation, and smoking cessation, there was not enough evidence to establish causality. A deeper understanding of these associations may have important implications for the development of preventive strategies to enhance vascular health.

## Data Availability

The data source of arterial stiffness during the current study is available in the IEU OpenGWAS project Trait: (https://gwas.mrcieu.ac.uk/datasets/ebi-a-GCST008403/). And the data source of smoking and alcohol consumption is from the GWAS and Sequencing Consortium of Alcohol and Nicotine Use. The final SNP information for exposures is included in this published article and its supplementary material.
